# Angiotensin-converting enzyme insertion/deletion gene polymorphisms and the risk of glioma in an Algerian population

**DOI:** 10.11604/pamj.2019.32.197.15129

**Published:** 2019-04-23

**Authors:** Ikram Hana Benenemissi, Karima Sifi, Lakhder Khalil Sahli, Ouarda Semmam, Noureddine Abadi, Dalila Satta

**Affiliations:** 1 Department of Animal Biology, Faculty of Life and Natural Sciences, Molecular and Cellular Biology Laboratory, University of Constantine 1, Constantine, Algeria; 2 Department of Biochemistry, Ben Badis University Hospital, Biology and Genetics Research Laboratory, Faculty of Medicine, University of Constantine 3, Constantine, Algeria; 3 Department of Neurosurgery, Regional Military Hospital of Constantine (HMRUC), Constantine, Algeria

**Keywords:** Angiotensin-converting enzyme, gene polymorphism, insertion/deletion, Glioma

## Abstract

**Introduction:**

Just recently, it has been established that the angiotensin-converting enzyme (ACE) insertion/deletion (I/D) polymorphism is linked to the pathogenesis and to the evolution of human cancers. Therefore, the present study was concerned with the investigation of an eventual association between glioma and I/D polymorphism of the ACE gene.

**Methods:**

The expression of ACE gene was detected by polymerase chain reaction restriction fragment length polymorphism (PCR-RFLP) analysis in 36 Algerian patients with glioma and 195 healthy controls.

**Results:**

In glioma cases, allelic frequencies and genotypes distribution of the ACE I/D polymorphism were different from controls cases. ACE DD genotype were highly presented in glioma cases (63.9%) than controls (33.8%) and conferred 3.64-fold risk for predisposition in glioma cases (vs ID genotype, p<0.001). Recessive model (ACE II + ID genotypes vs DD) was associated with a 72% reduced risk of glioma (OR = 0.28, 95% CI: 0.13-0.60, p <0.001). Per copy D allele frequency was found higher in glioma cases (79.2%) than in controls (63.3 %), OR = 2.20, 95% CI: 1.20 - 4.03, p = 0.009.

**Conclusion:**

The obtained data showed that the presence of the D allele might be a risk factor for the development of glioma. Further studies considering different ethnic groups with large samples are required to confirm this finding.

## Introduction

Gliomas are the most common primary intraparenchymal tumors of the central nervous system (CNS) with a very poor prognosis. They represent approximately 26% of all primary brain and other CNS tumors and 81% of the malignant tumors. Glioblastoma accounts for the majority of gliomas (56.6%). Relative survival estimates for glioblastoma are quite low with only 5.6% of patients who survived five years post diagnosis [1] . Historically gliomas have been classified according to the World Health Organization (WHO) criteria that are determined by histopathological examinations such as nuclear atypia, cellular pleomorphism, mitotic activity, vascular thrombosis, microvascular proliferation and necrosis [2]. Recently, in May 2016, a WHO reclassification of CNS tumors was established where the main change was the use of molecular parameters for diagnosis. Major restructuring of diffuse gliomas with incorporation of genetically defined entities was proposed [3]. This new classification is appropriate and makes a significant advance, because therapeutic targets are greatly dependent on the molecular mechanisms of gliomas, and tumors diagnosed in the same group have different gene expression profiles identified by large-scale genomic DNA analyses, including gene expression profiling, genome-wide association studies and single-nucleotide polymorphism analysis [4]. Furthermore, several environmental factors such as therapeutic ionising radiation, electrical or electronics jobs, long-term use of wireless phones, etc. have been correlated with an increased risk of developing gliomas. Genetic factors, using three signaling pathways: p53, retinoblastoma (RB), and receptor tyrosine kinase (RTK) play crucial roles in glioma development, particularly mutations in isocitrate dehydrogenase 1 and 2, 1p and 19q co-deletion [5-7]. The identification of genetic, epigenetic and transcriptional abnormalities in the various types of gliomas helps understanding the pathogenesis, predicts prognosis and response to therapy and may serve as diagnostic markers. Angiotensin-converting enzyme (ACE) is a zinc metallopeptidase that converts the inactive decapeptide angiotensin I to the vasoactive angiotensin II while inactivating an angiogenic agent, namely the vasodilator peptide bradykinin.

ACE is a member of the renin-angiotensin system (RAS) expressed in many tissues. Several findings have shown that the ACE inhibitors may possibly reduce the incidence of cancer and may protect against it [8, 9]. Their effect on tumor cells can be attributed to their reducing cell proliferation and migration, inflammation and angiogenesis [8, 9]. Other research works reported an inhibition of the cellular matrix metalloprotease activity and a reduced expression of vascular endothelial growth factor (VEGF) by ACE inhibitors [9, 10]. The ACE gene is located on the band 17q23 of the 17^th^ chromosome in humans. It is characterized by a major polymorphism exhibiting presence (I allele, insertion) or absence (D allele, deletion) of a 287-base pair Alu repeat sequence in intron 16 of this gene [9-11]. The ACE I/D polymorphism is correlated to the ACE plasma concentrations. In patients with DD genotype, ACE's plasma and tissue levels are about twofold higher compared to those with II genotype, whereas the ID genotype indicates an intermediate level [9-12]. Recently, the ACE I/D polymorphism was linked to the pathogenesis and progression of several cancers such as lung, digestive, breast, laryngeal, prostate cancers and last in glioma [9, 13, 14]. However, the results were not often reproducible, due to a genetic heterogeneity factor between different populations. In a Dutch population based prospective cohort study that included 6670 participants with ACE genotype, 730 incident cancer cases occurred during a mean follow-up time of nearly ten years [15]. Carriers of the high-activity genotype DD, with higher levels of ACE, had an increased risk of breast cancer compared with low-activity II/ID genotype carriers (hazard ratio (HR) = 1.47; 95% CI: 1.05-2.04), but no association was demonstrated for other cancers (prostate, lung and colorectal cancer). DD carriers who were exposed to long-term and high-dose of RAS inhibitors were at lower risk for cancer (HR = 0.28; 95% CI: 0.10-0.79). Short-term, high-dose users were at risk for colorectal cancer progression in the II/ID stratum (HR = 3.83; 95% CI: 1.67-8.79). In a meta-analysis including 6 studies, carriers of the ACE II genotype appeared to be protected from gastric cancer (OR 0.54-0.63, p = 0.01-0.02), regardless of ethnicity or gastric tumor type [16]. In a German study 88 samples from patients with early gastric cancer, which were obtained during gastric surgery, were compared with 145 blood samples from healthy controls [17]. The DD genotype was found significantly more often in the cancer group than in the healthy participants. The II genotype was associated with a significantly lower risk of gastric cancer than the DD genotype (OR=0.20, 95% CI: 0.08-0.54). Therefore, the aim of the present study was to evaluate the potential association between ACE I/D polymorphism and glioma in Algerian population and consequently on its potential role for developing therapeutic drug targets for this cancer.

## Methods

**Subjects:** the study was carried out in Constantine Ben Badis Hospital, involving 36 patients who were diagnosed as having glioma during 2016-2017 as well as 195 healthy controls which were matched with the glioma cases for age and sex. The controls were selected randomly from healthy individuals without any cancer history and any diseases that might have an association with the ACE I/D polymorphism, such as hypertension, coronary artery disease, diabetes mellitus and inflammatory diseases.

**DNA extraction and determination of the ACE I/D polymorphism:** a volume of 10 ml of peripheral venous blood was extracted from all subjects and the collected samples were put into BD Vacutainer spray-coated K2 EDTA. Standards methods were followed for extraction of genomic DNA from leukocytes [18]. A polymerase chain reaction restriction fragment length polymorphism (PCR-RFLP) assay was applied to assess the ACE I/D genotypes which was classified as II, ID and DD. Based on the GenBank reference sequence, the PCR primers were as follows: forward-5'-CTGGAGACCACTCCCATCCTTTCT-3' and reverse-5'-GATGTGGCCATCACATTCGTCAGAT-3'. PCR reactions were performed in 50 μl reaction volumes with 80 pmol of each primer, 25 pmol of dNTP (Biomatique), 1X of 10X buffer (Biomatique), 1.5 mm of MgCl_2_ (Biomatique), 0.5 U of DNA taq polymerase (Biomatique). DNA amplification was performed as follows: initial denaturation step for 1min at 94°C, followed by 30 cycles of denaturation; each step contained denaturation step at 95°C for 30 seconds, annealing step at 65.8°C for 30 sec and final extension at 72°C for 1 min. Finally, DNA underwent a final extension step at 72°C for 1 min. The PCR products had two weights of 490 and 190 bp for insertion and deletion, respectively. The products were first visualized by electrophoresis in a 2% agarose gel with ethidium bromide and finally in UV.

**Statistics:** descriptive statistics were performed in terms of means and percentages. The bivariate analysis used contingency tables and chi-square test to compare the genotype distribution between the two groups and crude odds ratio were calculated within a 95% confidence interval while p-value was calculated by Fisher's exact test. P values were considered statistically significant if they were below 0.05. Statistical analysis was performed using PRISM software, version 5.5.

**Ethical considerations:** the study was approved by an official review board and a declared consent was signed by patients themselves, their parents or legal tutors before blood sampling.

## Results

Characteristics of the considered population are shown in Table 1. Glioma cases and controls did not differ regarding gender (p = 0.37) or age (p = 0.83). Mean age was similar among patients and controls with 44.70 ± 3.33 and 42.61 ± 5.36 years, respectively. In glioma cases, allelic frequencies and genotype distribution of the ACE I/D polymorphism were different from controls cases as shown in Table 2. The frequency of the ACE DD genotype was higher in glioma patients than in controls with 63.9% and 33.8%, respectively and conferred 3.64-fold risk for predisposition in glioma cases (vs ID genotype, p<0.001). ID genotype was found with 30.6% and 59.0% in gliomas and in controls, respectively, II genotype with 5.6% and 7.2%, in gliomas and in controls, respectively. Recessive model (ACE II + ID genotypes vs DD) was associated with a 72% reduced risk of glioma (OR = 0.28, 95% CI: 0.13-0.60, p<0.001) (Table 2). Likewise, the frequency of the allele D was higher in glioma cases than in controls with 79.2% and 63.3%, respectively, OR = 2.20, 95% CI: 1.20 - 4.03, p = 0.009. The frequency of ACE I allele was 20.8% and 36.7 % in patients and in healthy controls, respectively.

**Table 1:** Characteristics (mean ± SD or %) of Algerian population with glioma and healthy controls

	Cases n (%)	Controls n (%)	*p*
Total	36 (100)	195 (100)	
Age (years)	44.70 ± 3.33	42.61 ± 5.36	0,83
Gender			
Males	23 (63.9)	109 (55.9)	0.37
Females	13 (36.1)	86 (44.1)	
Smoking status			
Non-smoking	30 (83.3)	189	0.007
Active smoking	5 (13.9)	5	0.002
Passive smoking	1 (2.8)	1	0.18
Family history of glioma	7 (19.4)	0 (0)	< 0.001

**Table 2:** ORs and 95% CIs of the association between ACE I/D polymorphism and glioma risk

Model	Cases (n)	Controls (n)	OR	*p*
Homozygous co-dominant: DD vs II				
DD	23	66	2.43 (0.51-11.55)	0.24
II	2	14		
Heterozygous co-dominant: ID vs II				
ID	11	115	0.66 (0.13 -3.33)	0.62
II	2	14		
Heterozygous co-dominant: DD vs ID				
DD	23	66	3.64 (1.67-7.94)	<0.001
ID	11	115		
Dominant model: (DD + ID) vs II				
DD + ID	34	181	1.31 (0.28-6.04)	0.7
II	2	14		
Recessive model: (II + ID) vs DD				
II + ID	13	129	0.28 (0.13-0.60)	<0.001
DD	23	66		
Allele model				
D	57	247	2.20 (1.20-4.02)	0.009
I	15	143		

## Discussion

As mentioned above glioma is the most common malignant tumor in the central nervous system which may be caused by many environmental, genetic or other factors. Multiple genetic and cytogenetic alterations have been identified in gliomas with a regularly increasing number. Several major molecular alterations have been found, such as IDH1/IDH2 (Isocitrate dehydrogenase) mutations in diffusely infiltrating gliomas, mutations of TP53 (tumor suppressor protein 53) and ATRX (Alpha-thalassemia/mental retardation syndrome X-linked) in astrocytomas, 1p/19q co-deletion in oligodendrogliomas, mutations of TERT (telomerase reverse transcriptase) promoter in oligodendrogliomas and IDH wild-type glioblastomas, and mutations or fusions of BRAF in circumscribed astrocytomas [19]. Identification of those and several other genetic abnormalities is very important and may help to determine appropriate treatment and to predict prognosis. Otherwise, recently, many mechanisms to explain tumorigenesis known as the “hallmarks” of cancer have been proposed [20] and they are connected to many biochemical pathways including the RAS whose role in CNS tumors has led to an increasing interest among researchers. Classically, the RAS has been studied as a fundamental component of cardiovascular homeostasis, playing a critical role in cardiovascular system, balance of water and electrolytes and cell growth [8, 21, 22]. However, RAS also is expressed in several tissues and organs (liver, kidneys, pancreas, reproductive organs, and brain). Some of these local effects are related to carcinogenesis and gliomagenesis [8]. The perturbation of RAS components plays a significant role in the proliferation, angiogenesis, and invasion of these tumors. Angiotensin II can stimulate tumor neovascularization [23], and over expressions of ACE and angiotensin II type 1 receptor have been associated with tumor growth, metastasis, and progression [24-26]. The ACE gene I/D polymorphism has been linked to many cancers with a very few researches on glioma. The present study was interested in potential association between ACE I/D polymorphism and glioma in Algerian population. The main result indicated a strong association between this polymorphism and the risk of glioma. A recessive model was associated with a 72% reduced risk of glioma (p<0.001). Likewise, the DD genotype predisposed risk of glioma by more than 3-fold i.e. 63.9 and 33.8% in glioma cases and controls, respectively (p<0.001), indicating the potential of ACE I/D polymorphism as a predictive marker in glioma. We also found that the risk conferred by a per copy allele D is 2.2-fold for glioma cases. Frequency of D allele was higher in glioma cases than in controls (79.2% and 63.3%, respectively, p = 0.009). Those results are supported partly by the first report describing the potential association between ACE I/D polymorphism and glioma in Chinese population. In this study, glioma cases had a significantly higher frequency of DD genotype (OR = 1.61, 95% CI = 1.12, 2.32; p = 0.01) than controls [13]. But the frequency of D allele was similar in glioma cases and in controls (30.9% vs 28.3%, p = 0.1)

In another recent study in Indian population: ACE DD genotypes were highly presented in glioma cases compared to controls with 26.8% and 10.6%, respectively (p<0.0001) and conferred 5-fold risk for predisposition in glioma cases. Per copy D allele frequency was found higher in glioma cases than in controls (54% and 25%, respectively, p<0.0001) [14]. No similar studies were found in North Africa. For instance, a Moroccan study summarized 3 research works with 65, 62 and 34 patients, on the frequency of altered genes in patients with glioblastoma, where only alteration of IDH1/2, p53 and EGFR expression were analysed [27]. The frequency of these gene mutation in Moroccan population was similar to those reported from other populations. In Tunisian population with glioma, genetic analysis in 110 cases of glioma assessed 10q LOH including PTEN as the most frequent chromosome alteration [28]. Moreover, the impact of studies on ACE I/D polymorphism in glioma was also therapeutic. The results in the Rotterdam study concerning a prospective cohort with 7983 participants, showed that RAS inhibitors seemed to protect against cancer in patients with the ACE DD genotype [15]. Indeed, ACE's plasma and tissue levels were higher in subjects with DD genotype, compared to those with II or ID genotypes and its activity can be blocked by ACE inhibitors [9, 12]. Their effect on tumor cells could be attributed to their reducing cell proliferation and migration, inflammation and angiogenesis, inhibition of the cellular matrix metalloprotease activity and a reduced expression of VEGF (8-10). Finally, it should be recognized that the current study was the first one in the middle east and north Africa (MENA) region, that had investigated the association between ACE I/D polymorphism and glioma, bearing in mind the potential application of the RAS components as biomarkers or treatment targets in glioma. However, it should be precised that only the Eastern population of Algeria was concerned by the present study which then cannot be considered as fully representative of the entire country population. Although the size of the population was small due to the fact that this pathology is relatively rare in Algeria, with about 1 percent only of all cancers (unpublished data from the Constantine University Hospital), the number of cases was consistent for a statistical analysis to obtain useful results and to define the orientation to be given to further studies. Nevertheless, a study with a large sample and a follow-up study to analyze glioma overall survival by ACE I/D genotype are necessary.

## Conclusion

The obtained data suggest that the ACE I/D polymorphism could be a risk factor for glioma. However, this has to be confirmed with additional studies considering large samples of different ethnical groups.

### What is known about this topic

- The ACE gene I/D polymorphism has been linked to many cancers;
- Recently, only 2 reports had shown the relationship between this polymorphism and glioma.

### What this study adds

- The ACE I/D polymorphism could be a risk factor for glioma or predictive marker;
- Renin-angiotensin system blockade may be used in a future as treatment or preventing glioma.

## Competing interests

The authors declare no competing interests.
